# A molecular mechanism of symmetry breaking in the early chick embryo

**DOI:** 10.1038/s41598-017-15883-8

**Published:** 2017-11-17

**Authors:** Clemente F. Arias, Miguel A. Herrero, Claudio D. Stern, Federica Bertocchini

**Affiliations:** 1 Departamento de Matemática Aplicada, Facultad de Matemáticas, and Universidad Complútense de Madrid, Madrid, Spain; 2 Grupo Interdisciplinar de Sistemas Complejos (GISC), Universidad Complútense de Madrid, Madrid, Spain; 3 Department of Cell and Developmental Biology, University College London, London, UK; 4 Instituto de Biomedicina y Biotecnología de Cantabria (IBBTEC)-CSIC-Universidad de Cantabria, Santander, Spain

## Abstract

The first obvious sign of bilateral symmetry in mammalian and avian embryos is the appearance of the primitive streak in the future posterior region of a radially symmetric disc. The primitive streak marks the midline of the future embryo. The mechanisms responsible for positioning the primitive streak remain largely unknown. Here we combine experimental embryology and mathematical modelling to analyse the role of the TGFβ-related molecules BMP4 and Vg1/GDF1 in positioning the primitive streak. *Bmp4* and *Vg1* are first expressed throughout the embryo, and then become localised to the future anterior and posterior regions of the embryo, where they will, respectively, inhibit or induce formation of the primitive streak. We propose a model based on paracrine signalling to account for the separation of the two domains starting from a homogeneous array of cells, and thus for the topological transformation of a radially symmetric disc to a bilaterally symmetric embryo.

## Introduction

How do vertebrate embryos break their initial radial symmetry and establish a midline as the axis of bilateral symmetry? In amphibians and fishes, the whole embryo is initially patterned by antagonistic gradients of BMP (ventrally) and Wnt/Nodal/Activin and BMP antagonists (dorsally)^1–3^. The difference between dorsal (where gastrulation starts) and the opposite side is set up by localization of maternal determinants. However, in amniotes (birds and mammals, and presumably also reptiles) zygotic transcription starts very early, allowing embryonic regulation until quite late. For example, a chick embryo at the 20,000–50,000 cell stage can be divided into 4 or more fragments, all of which can initiate the formation of a primitive streak^4,5^. These observations suggest that localization of maternally produced molecules cannot be the sole determinant of bilateral symmetry or the position of the embryonic axis in amniotes. In the early chick embryo, the posterior marginal zone (adjacent to where the primitive streak will form) expresses the TGFβ superfamily member *Vg1* ^6–10^, which is both sufficient^6–10^ and necessary^11^ for primitive streak formation. The opposite (anterior) margin expresses the transcription factor *Gata2*, which appears to act as a weak inhibitor of primitive streak formation. Previous experiments suggested that *Gata2* and *Vg1* transcription is regulated independently at the opposite ends of the embryo, which led to the proposal of a Global Positioning System (GPS) to pattern the whole embryo^11^.

What is the molecular nature of this GPS? Gata2 knockdown causes downregulation of *Bmp4* expression, consistent with an involvement of BMP in positioning the primitive streak^12^. This suggests that BMP signalling might constitute one of the elements in the embryo GPS. To explore this possibility, we examined the earliest expression of *Bmp4* and *Vg1*. *In situ* hybridization on embryos earlier than stage X EG&K^13^ reveals that both *Bmp4* and *Vg1* are expressed ubiquitously (Supplementary Figure SF1 A-I). By stage X, the expression domains of these genes separate to opposite poles of the blastodisc (Supplementary Figure SF1 J-P). This raises the question of how this segregation takes place.

**Figure 1:**
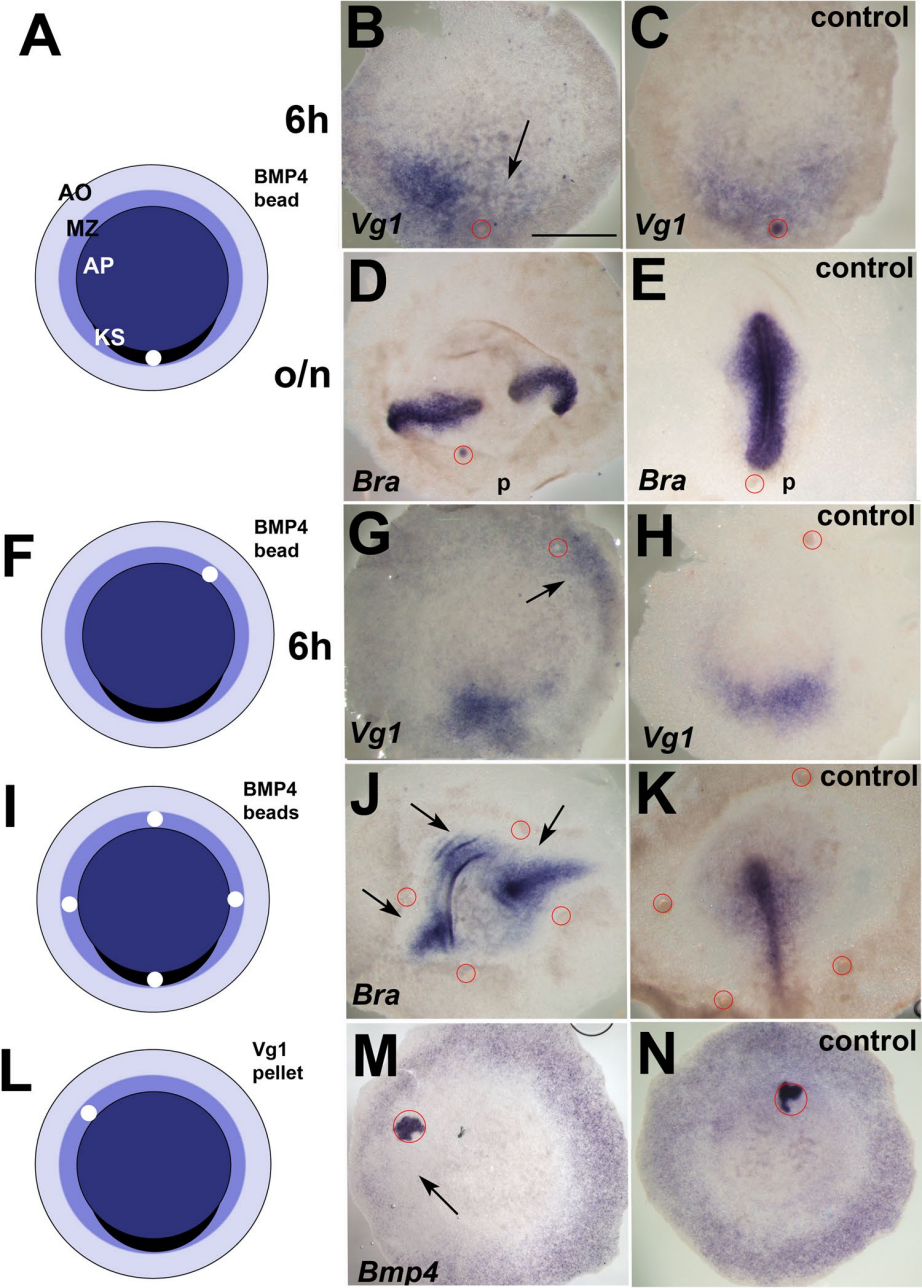
BMP4 and Vg1 dynamics in the early embryo. (**A**–**E**) Graft of BMP4-bead in the posterior marginal zone (**A**) inhibits *Vg1* expression (**B**) and axis formation as indicated by *Brachyury* (*Bra*) expression (**D**) (**C**, **E**: controls). (**F**–**H**) Anterior BMP4-bead (**F**) induces *Vg1* expression (**G**, arrow, **H**, control). (**I**–**K**) Multiple BMP4-conjugated bead graft (**I**) induces multiple axes (*Bra* expression) (**J**, arrows) (**K**, control). (**L**–**N**) Vg1 misexpression anteriorly (**L**) causes *Bmp4* downregulation (**M**), (**N**, control). Red circle: BMP4 bead in all figures except (**M**,**N**), where it indicates the pellet of COS cells. Posterior (p) to the bottom. Scale bar: 1 mm.

In order to understand the role of BMP4 in positioning the primitive streak, and BMP4 relation with Vg1 we analysed the effects of ectopic BMP4 in different regions of the embryo. A bead of BMP4 placed in the posterior marginal zone (Fig. 1A) causes downregulation of *Vg1* (23/26, control: 0/10) (Fig. 1B,C). *Vg1* downregulation was paralleled by inhibition of primitive streak formation: in 42/49 embryos incubated overnight after a posterior graft of a BMP4 bead, the primitive streak failed to form near the bead (as previously reported^12^), but two streaks arose from lateral positions (control: 0/32) (Fig. 1D,E). Paradoxically, grafts of a bead of BMP4 in the anterior/lateral marginal zone (Fig. 1F) caused upregulation of *Vg1*. In 17/34 embryos *Vg1* expression was upregulated within 6 hours (control: 0/33) (Fig. 1G,H and Supplementary Figure S2). Simultaneous inducer and inhibitor effects of BMP4 on *Vg1* were evident even in the same embryo (Supplementary Figure SF 2C). We grafted four BMP4 beads in the marginal zone (as shown in Fig. 1I). 9 out of 12 embryos developed multiple primitive streaks, spaced between the beads (control: 0/12) (Fig. 1J,K). The paradoxical opposite effects elicited by BMP4 on the anterior and posterior parts of the early embryo on *Vg1* expression support the idea that BMP4 is part of the GPS, and is thus involved in positioning the primitive streak. If BMP4 is indeed part of the GPS system that positions Vg1, is the converse also true? To test this, we grafted a pellet of Vg1-transfected cells onto the anterior marginal zone (Fig. 1L). In 7/12 embryos, *Bmp4* expression was downregulated (control: 0/12) (Fig. 1M,N).

Taken together, the above experiments suggest that BMP4 and Vg1 can inhibit each other’s expression when misexpressed in each other’s domain, but overexpression of BMP4 anteriorly paradoxically induces *Vg1*. What mechanisms could account for this? Opposite effects of BMP4 on *Vg1* in different regions of the embryo can hardly be explained by assuming any prior difference between cells in anterior and posterior regions. In order to get insights on how a homogeneous field of cells can give rise to a distinct pattern which results in an antero-posterior symmetry we formulated a mathematical model of BMP4 and Vg1 interactions.

Mathematical modelling has been widely used to explore self-regulated pattern formation in biological systems. The most commonly used models are based on reaction-diffusion (RD) mechanisms^14,15^ based on long-range diffusion of morphogens that can generate patterns at long range, and have recently been used to understand how patterns such as the formation of structures like rugae in the hard palate^16^ and digit patterning^17^ occur in mouse. The standard RD approach postulates that the spatial distribution of molecular signals (morphogens) is determined by direct interactions among them, and by their diffusion across a given domain^14,15^. However, RD systems are not appropriate to model interactions in the early chick blastoderm, because a) the embryo at this stage is a very large flat disk (about 3 mm diameter), just one cell thick, suspended between two large volumes of fluid (albumen dorsally, yolk ventrally), with virtually no extracellular space to establish a stable gradient based on diffusion; b) the source of Vg1 is comparatively far away from the opposite pole of the embryo; c) free, extracellular diffusion cannot provide an efficient physical mechanism for anterior and posterior embryonic regions to interact via diffusive chemical signals. A more parsimonious explanation could involve paracrine (local) signalling between nearby cells^18^. Both BMP4 and Vg1 are secreted signalling proteins that interact with specific membrane receptors, located in the same cell or in nearby cells. Therefore, they do not need to diffuse across particularly large distances within the embryo to be fully functional. We propose a model based on the idea that BMP4 and Vg1 interact by a short-range paracrine activity, whereby the signalling process is maintained by signal renewal triggered by signal-receptor interactions, rather than following from collision-like chemical reactions outside the cell (see scheme in Supplementary Material SM1 for details). An algorithm that only requires two transcription factors, labelled F_B_ and F_V_, mediates feedback interactions between BMP4 and Vg1 in neighbouring cells. Pairwise interactions between BMP4, Vg1, F_B_ and F_V_ are represented by means of Hill-type equations (see SM1), which have been used in a variety of genetic systems^19,20^, because they can describe activation and inhibition mechanisms in a straightforward manner.

**Figure 2:**
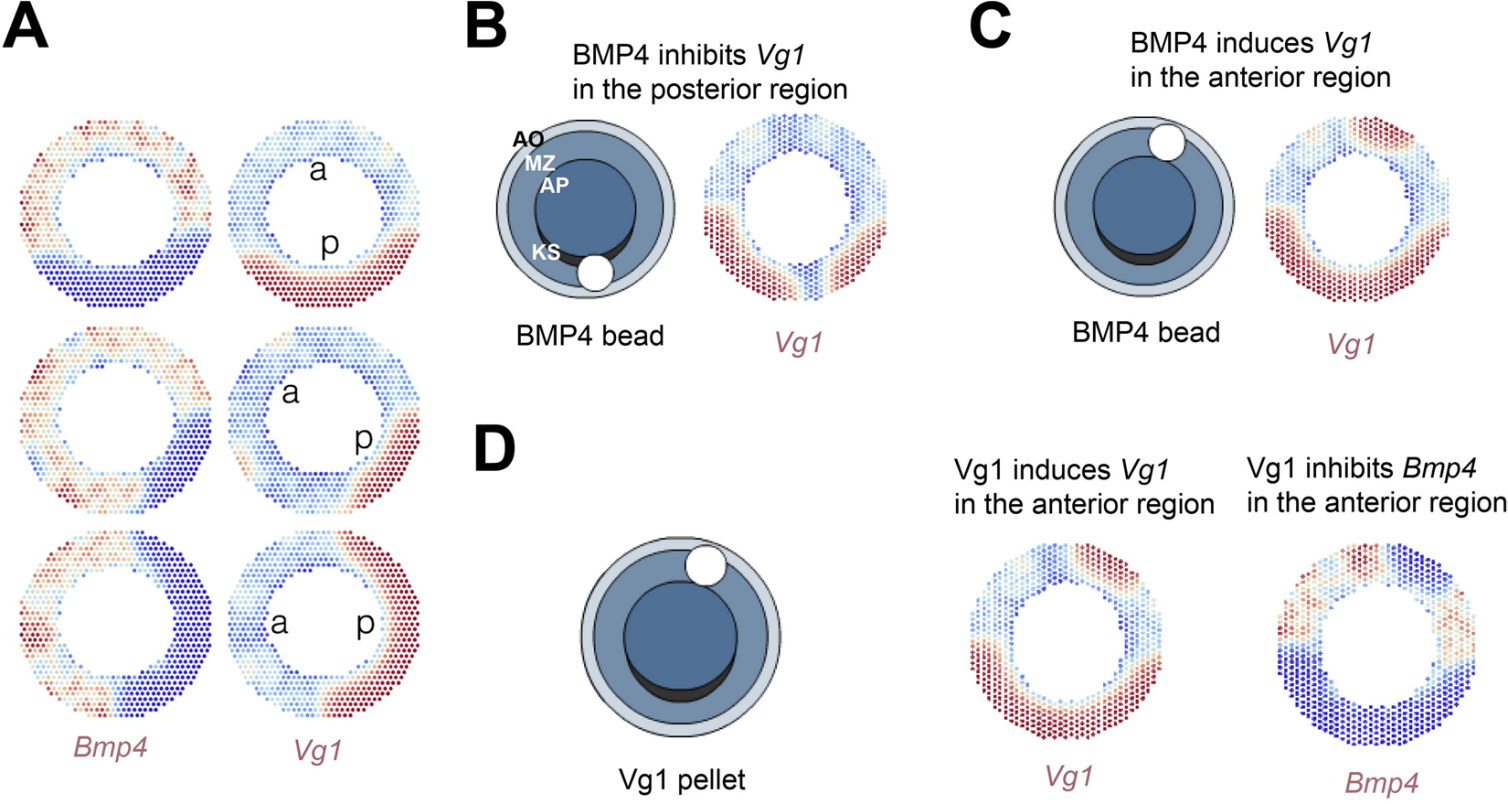
Numerical simulations of the model describing Bmp4 and Vg1 dynamics. (**A**) In agreement with experimental results, two opposite gradients of *Bmp4* and *Vg1* form in the embryo starting from a homogeneous initial situation. The images correspond to numerical simulations starting with three different initial conditions. (**B**) Ectopic expression of BMP4 posteriorly inhibits *Vg1*. (**C**) In contrast, ectopic BMP4 anteriorly induces local *Vg1*. (**D**) Vg1 anteriorly induces its own expression and inhibits *Bmp4*. Blue and red indicate lower and higher levels of expression, respectively. The values of parameters used in these simulations are shown in SM2.

With these elements we implemented an agent-based model in which the same functional relations between BMP4 and Vg1 operate in each individual cell of the marginal zone (see SM2, with diagram in A5). Starting from a ubiquitous, uniform expression pattern in a homogeneous field of cells proposed to be identical, the model can generate a coherent collective behaviour, leading to segregation of BMP4 and Vg1 to opposite poles of the embryo (Fig. 2A). Importantly, no initial bias is necessary to induce the breaking of radial symmetry of the embryo. In this respect, all cells are proposed to behave according to the same interactions as described in SM2, and simulations were performed starting from initially homogeneous values. Therefore, the resulting macroscopic pattern is an emergent property of the model.

Next, we tested if the model can reproduce the paradoxical effects of ectopic expression of BMP4 in anterior and posterior regions of the embryo. We postulate that initially, interactions between BMP4 and Vg1 (described in SM2) take place continuously in every cell in the epiblast, irrespective of their location in the embryo. Indeed, the model reproduces our experimental results: in normal embryos, the domains of expression of BMP4 and Vg1 segregate to opposite sides of the blastoderm. In experimental embryos, the model reproduces the findings that overexpression of BMP4 posteriorly inhibits *Vg1* expression (Fig. 2B), whereas anterior misexpression of BMP4 paradoxically induces *Vg1* near the site of overexpression (Fig. 2C). The model also predicts the previously reported observation^8^ that ectopic expression of Vg1 anteriorly induces *Vg1* expression, and also that it should inhibit *Bmp4* expression there (Fig. 2D).

**Figure 3:**
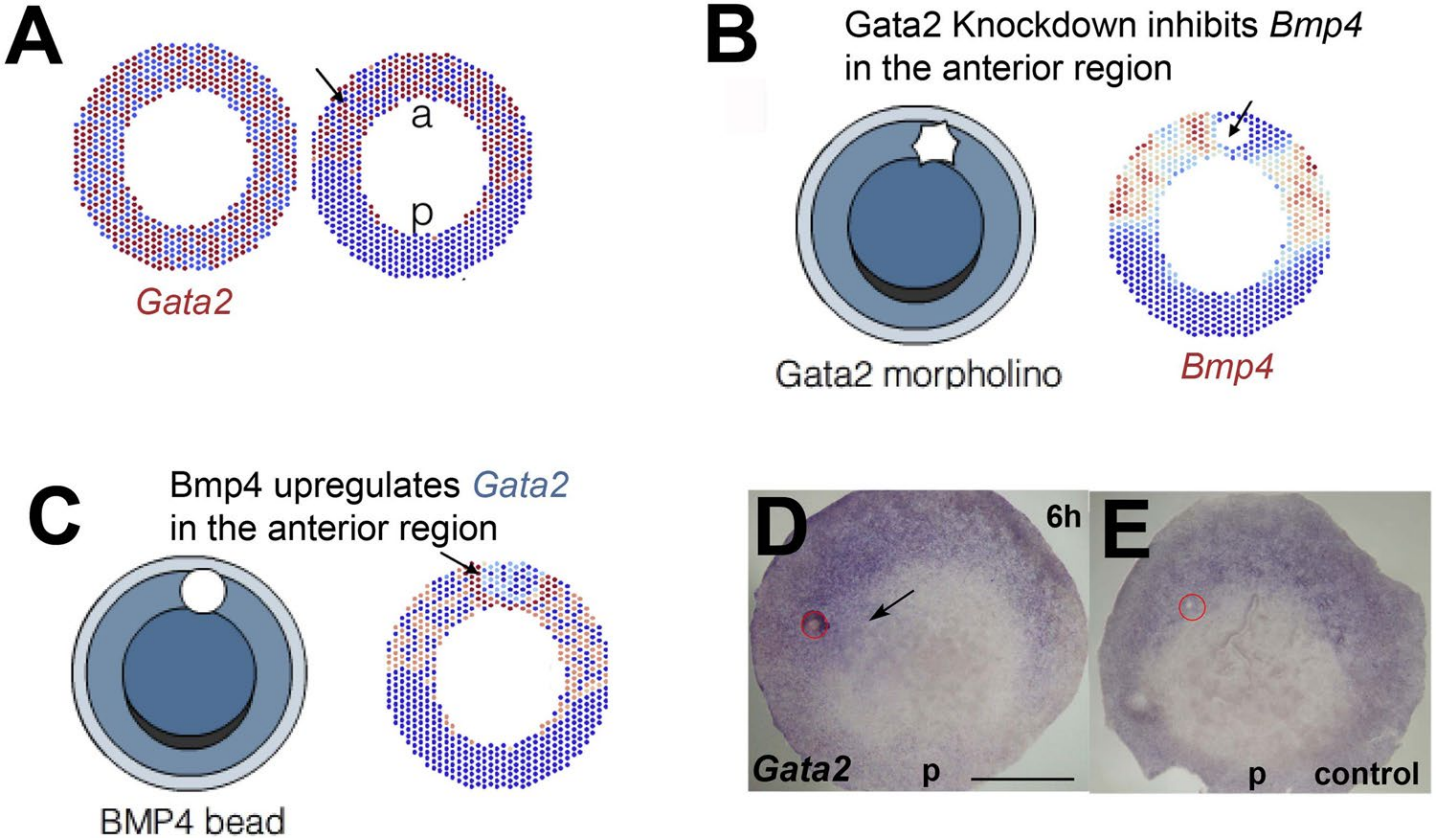
Gata2 is a potential candidate for factor F_B_. (**A**) Initial and final distribution of Gata2 as predicted by the model. Left: *Gata2* is initially homogeneously distributed. Right: After model simulation, *Gata2* appears segregated to the anterior region. (**B**) Numerical simulations of Gata2 knockdown produce a local *Bmp4* downregulation. (**C**–**E**) Numerical simulation of ectopic BMP4 anteriorly results in local *Gata2* upregulation (**C**), confirming the experimental result (**D**, arrow) (**E**, control). Posterior (p) to the bottom. Red circle: BMP4 (**D**) or control (**E**) bead. Scale bar: 1 mm.

The model invokes intracellular factors downstream of BMP4 and Vg1, F_B_ and F_V_, respectively. The transcription factor Gata2 is a candidate for F_B_. *Gata2* is expressed throughout the embryo before stage X, but eventually co-localizes in the future anterior region with *Bmp4* ^11^. The model reproduces these changes in *Gata2* expression (Fig. 3A). The model also predicts that Gata2 knockdown should result in *Bmp4* downregulation (Fig. 3B) and that anterior/lateral overexpression of BMP4 should increase *Gata2* mRNA levels (Fig. 3C). The first prediction agrees with published results^11^, so we tested the second by grafting a BMP4 bead in the anterior/lateral marginal zone. In 13/19 embryos *Gata2* expression was upregulated (control 0/14) (Fig. 3D). In the posterior region, Pitx2, a transcription factor that regulates *Vg1* expression^21^, could be a possible candidate for F_V_. *Pitx2* is slightly upregulated after BMP4 misexpression in the anterior/lateral marginal zone, with expression extending from the posterior region towards the bead (Figure SF3A,B, 6/18 embryos, control: 0/21, figure SF3C). However, upregulation of *Pitx2* after BMP4 misexpression is only seen in a proportion of the embryos and is weaker than that of *Vg1* in the same experimental conditions, keeping open the possibility that other molecules could fulfil the role of F_V_ (see ref.^21^ for genes expressed in the posterior region of the early embryo).

In this paper we described a self-organizing process to account for the breaking of radial symmetry and the establishment of bilateral symmetry in the avian embryo. The model (SM2) is based on a paracrine mode of action of BMP4 and Vg1, whereby a set of complex interactions with two intracellular factors, F_B_/Gata2 and F_V_ gives rise to a spatial pattern of expression that defines the anterior and posterior poles of the blastodisc and thereby anticipates the position of the primitive streak (see SM2). A role of BMP4 and Vg1/Nodal in early embryo polarity has been described in frog and fish^22,23^. However, to the best of our knowledge, this is the first description of dynamic interactions between BMP4 and Vg1 driving symmetry breaking that eventually results in primitive streak formation in amniotes. Interestingly, the model also suggests an explanation for the formation of twins (see SM2).

Could a similar mechanism work in mammals? In mouse, asymmetric Nodal activity drives movement of the distal VE towards the future anterior region, thus establishing the position of primitive streak formation^24^. *Bmp4* is expressed in the distal ring of extraembryonic epiblast in the early mouse embryo, and Bmp4 downregulation prevents gastrulation and mesoderm formation^25^. The latter effect is due to Bmp4 influence on Nodal antagonists in the VE^26^, which supports an inhibitory role in primitive streak formation. Onset of an ectopic primitive streak-like structure in the amnion, with Nodal upregulation, occurs in the Bmp-effector Smad5 knockout, suggesting the presence of a Bmp/Nodal antagonism^27^. Whether or not interactions between Bmp4 and Nodal determine the position of the primitive streak in the mouse embryo remains unknown.

Paracrine mechanisms could explain the emergence of self-organized spatial patterns in other multicellular patterning systems, such as small aggregates of mouse Embryonic Stem Cells^28^ or micro-patterned cultures of human Embryonic Stem Cells^29,30^. For example, in the latter case, cells confined to a disk shape self-organize into patterned concentric areas, reminiscent to a certain extent of the three concentric areas that define the early chick embryo (AO, MZ and AP). This suggests the potential deployment of similar mechanisms in the patterning of a group of cells arranged in a blastodisc shape. The model proposed here could help to design experiments to test whether similar mechanisms could operate under these conditions.

## Methods

### Embryos and manipulation

Fertile hens’ eggs were obtained from Granja Gibert (Spain) (Brown Bovan Gold) and staged in Roman numerals for pre-primitive streak stages^13^ and in Arabic numerals^31^ starting from stage 2, when the primitive streak appears. Embryos were cultured in modified New culture^32,33^. Pre-stage X embryos were collected using a manual retrieval method as previously described^34^. Cut-in-half experiment on stage X embryos was carried out as previously described^10^. No live vertebrates were used for the experiments.

### *In situ* hybridization

*In situ* hybridisation was carried out as described^35^ using the following probes: chick *Bmp4* ^36^, *Brachyury* ^37–39^, *Gata2* ^40^, *Vg1* ^7^, *Pitx2* ^21,41,42^.

### Gain-of-function experiments

To misexpress Vg1, we used a *Dorsalin-cVg1* expression construct^7^. We transplanted COS cells transfected with the construct of interest, and pellets of 1000 cells were generated from hanging drops and grafted into host embryos as previously described^7,8,12,43^. For misexpression via BMP4-conjugated to heparin beads (SIGMA), recombinant BMP4 (RD systems) was used at 15 μg/ml. Control beads were incubated in PBS.

## Electronic supplementary material

Supplementary Information
