# Supplementary material for: A molecular mechanism of symmetry breaking in the early chick embryo
Source: Sci Rep. 2017 Nov 17;7:15776. doi: 10.1038/s41598-017-15883-8 (PMC5694015; doi:10.1038/s41598-017-15883-8)
Supplement: Supplementary file 1 — Supplementary Information [file 41598_2017_15883_MOESM1_ESM.pdf]

Supplementary Information for: A molecular mechanism of symmetry breaking in the early chick embryo

Clemente F. Arias[*1,2], Miguel A. Herrero[1], Claudio D. Stern[3] and Federica Bertocchini[*4]

1 Departamento de Matemática Aplicada. Universidad Complutense de Madrid, Madrid, Spain
2 Grupo Interdisciplinar de Sistemas Complejos (GISC), Madrid, Spain
3 Department of Cell and Developmental Biology, University College London, London, UK
4 Instituto de Biomedicina y Biotecnología de Cantabria (IBBTEC)- CSIC-Universidad de Cantabria, Santander, Spain

E-mail CFA: tifar@ucm.es
E-mail FB: federica.bertocchini@unican.es

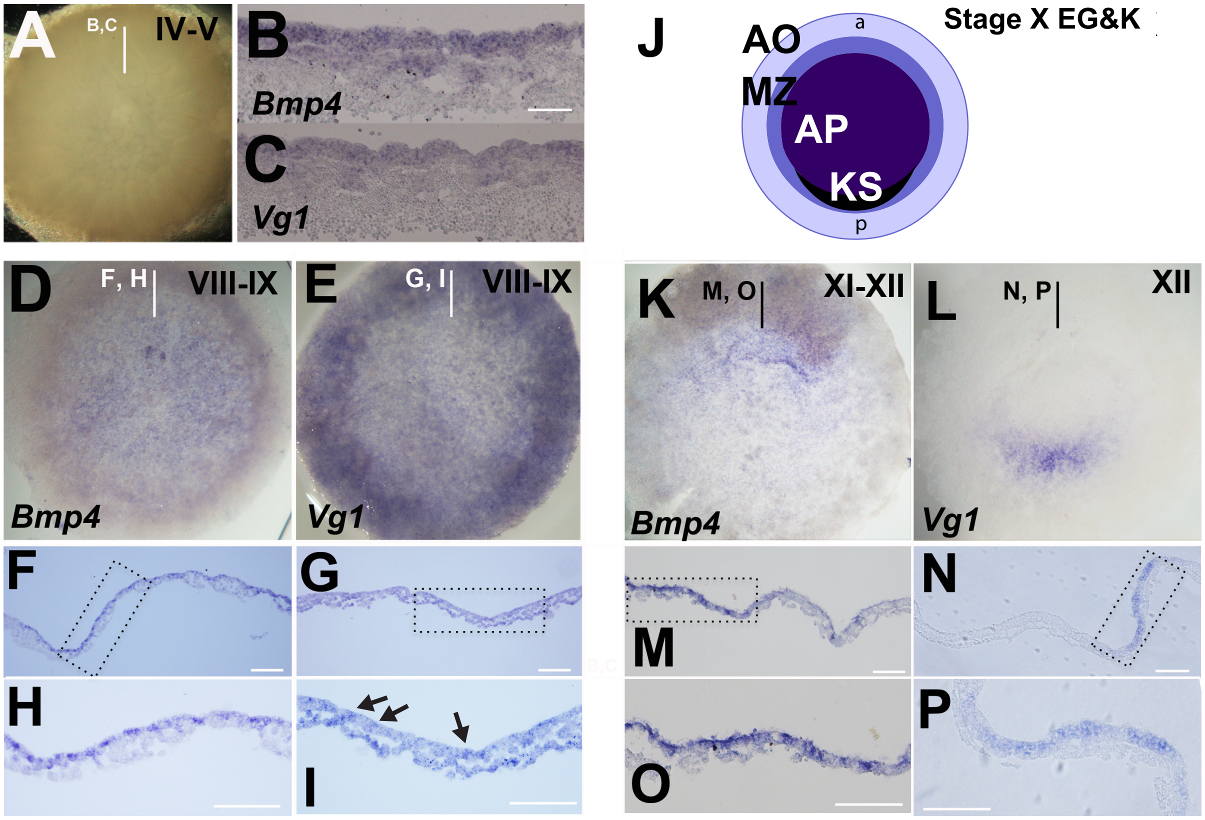

Supplementary Figure 1: ***Bmp4* and *Vg1* are ubiquitously expressed before stage X, when they localize on opposite poles of the blastodisc**. A-C. In situ hybridization on sections (B, C) of a stage IV-V EG&K chick embryo (A) shows ubiquitous expression of *Bmp4* (B) and *Vg1* (C). D-I, K-P. Whole-mount in situ hybridization for *Bmp4* (D, F, H, K, M, O) and *Vg1* (E, G, I, L, N, P) at stage VIII-IX and XI-XII chick embryos: both *Bmp4* and *Vg1* are ubiquitously expressed at stage VIII-XI (D-H and E-I, respectively), while they localize at opposite poles of the embryo after stage X (K-O and L-P, respectively). F-H, G-I, M-O, N-P are sections of the embryo in D, E, K, L, respectively, as indicated. J. Scheme of a stage X EG&K [1] chick embryo. The three concentric areas that constitute the epiblast are indicated with different colours: dark blue: Area Pellucida (AP); light blue: Marginal Zone (MZ); violet: Area Opaca (AP). Kollers sickle (KS) is indicated with a black semi-sickle area. a and p indicate anterior and posterior, respectively. Posterior to the bottom in K and L, to the right in M-P. Scale bar: 100 μm.

[1] H. Eyal-Giladi, S. Kochav, From cleavage to primitive streak formation: a complementary normal table and a new look at the first stages of the development of the chick. I. General morphology. *Developmental biology* **49**, 321-337 (1976);

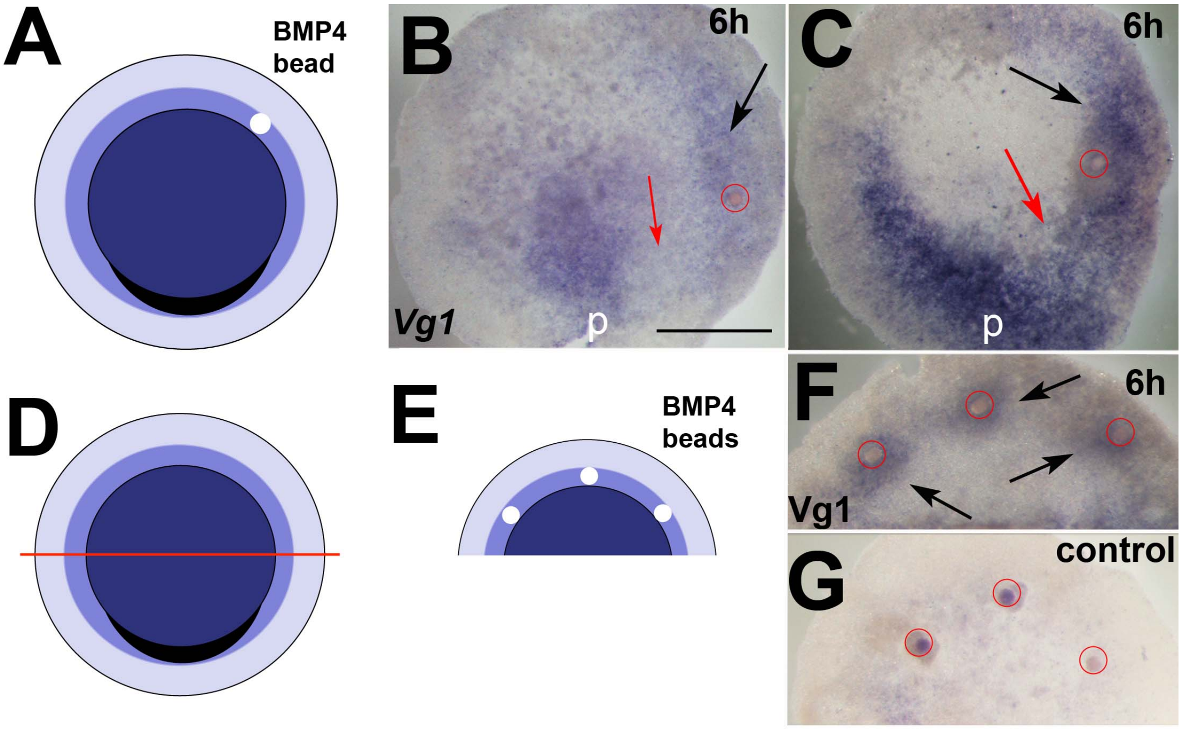

Supplementary Figure 2: **Bmp4 in the anterior/lateral region of the embryo induces Vg1 expression**. A-C. BMP4 bead in the anterior/lateral marginal zone (A) induces Vg1 expression after 6h of incubation (B, C black arrows); in some cases, BMP4 inhibitory and inducing effects on Vg1 occur in the posterior (red arrows) and anterior region (black arrows), respectively, of the same embryo. Posterior (p) to the bottom. D-G. In isolated anterior halves, which have been shown to regulate [2–5] three ectopic BMP4 beads induce Vg1 expression around the bead (arrows in F) after 6h of incubation (G, control beads). Scale bar: 1 mm.

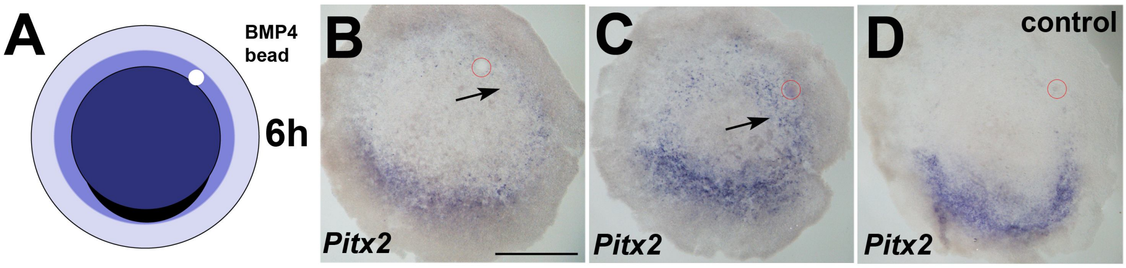

Supplementary Figure 3: **Bmp4 in the anterior/lateral region of the embryo faintly induces *Pitx2* expression**. A-C. BMP4 bead in the anterior/lateral marginal zone (A) induces a faint *Pitx2* expression after 6h of incubation (B, C black arrows). *Pitx2* expression extends from the posterior region towards the bead. (D) Control bead. Scale bar: 1 mm

Supplementary Material 1

Generic model of paracrine signaling

In this section we will describe the generic logic of the type of model presented in the text. In order to illustrate the main points of our approach, we will consider a simple, abstract example in which a paracrine protein labelled as **a** induces the expression of a second protein labelled as **b**. In the next section we will adapt this generic scheme to model the interactions between BMP4 and Vg1.

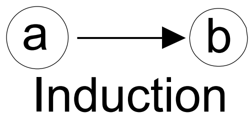

If protein **a** acts as a paracrine signal, then its effect on protein **b** should occur through the mediation of specific membrane receptors, and a series of intracellular factors, as shown in the following diagram:

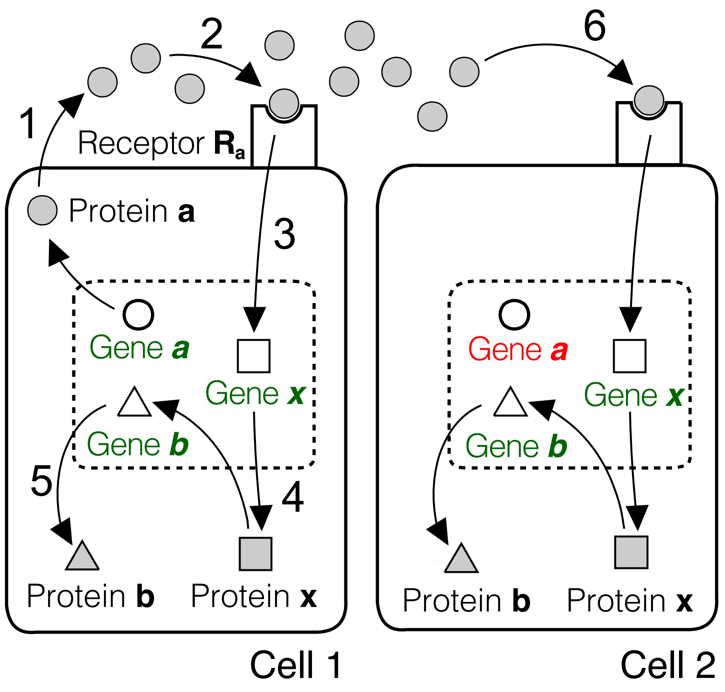

In this example, Cell 1 secretes protein **a** into the extracellular space (1). Once there, it can interact with its cognate membrane receptors $\mathbf{R_a}$ (2), which triggers the corresponding transduction pathway (3) and leads to the expression of transcription factor **x** (4). In turn,

the presence of factor **x** induces the expression of protein **b** (4). Protein **a** can also interact with receptors of neighbouring cells (6), leading to the expression of b, even if gene a is not active there. We remark that cells 1 and 2 in this example are identicalare identical as far as the interaction between **a** and **b** is concerned . However, both cells differ in the activation state of gene $\boldsymbol{a}$.

The role of transcription factor **x** as mediator of the effect of protein **a** on protein **b** can better described by explicitly considering it as induced by protein **a** and inducing the expression of protein **b**:

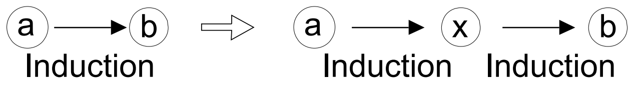

Hill-type equations

Qualitative relations such as induction of protein **b** expression by protein **a** as described above can be quantified by means of Hill type equations. These equations take the following expressions:

$$b'(t) = H^{+}(a) = \sigma \frac{a(t)^4}{\lambda^4 + a(t)^4} \qquad (\mathbf{a} \text{ induces } \mathbf{b})$$

$$b'(t) = H^{-}(a) = \sigma \frac{\mu^4}{\mu^4 + a(t)^4} \qquad (\mathbf{a} \text{ inhibits } \mathbf{b})$$

Where $a(t)$ and $b(t)$ represent the amount of proteins **a** and **b** expressed at time t respectively, and λ, μ and σ are positive parameters. The following figure displays the behavior of these equations. In activator-like dynamics the production of protein **b** increases with the concentration of protein **a**. Conversely, high amounts of **a** in inhibitory-like relations block the production of **b**.

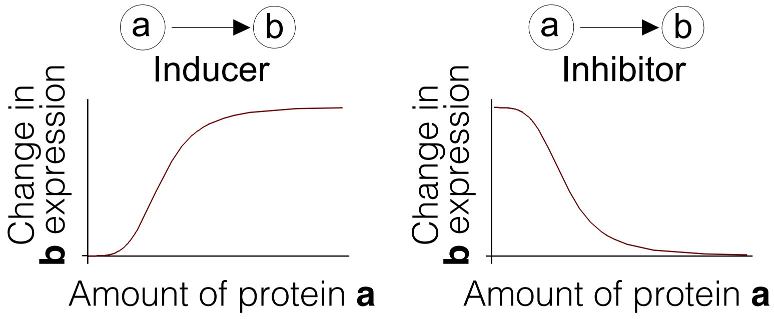

Dose-dependent effects can be modeled by combining the two previous equations:

$$b'(t) = H^{\pm}(a) = \sigma \frac{a(t)^4}{\lambda^4 + a(t)^4} \frac{\mu^4}{\mu^4 + a(t)^4} \qquad (\mathbf{a} \text{ activates } \mathbf{b} \text{ at intermediate doses})$$

In this case, intermediate concentrations of **a** induce **b** expression, while low and high concentrations of **a** inhibit **b** expression:

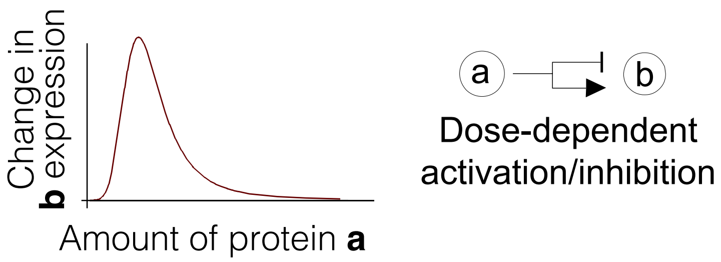

The range of concentrations of protein **a** that induce **b** expression can be modulated by means of an additional parameter τ:

$$b'(t) = H^{\pm}(a) = \begin{cases} \sigma \dfrac{(a(t) - \tau)^4}{\lambda^4 + (a(t) - \tau)^4} \dfrac{\mu^4}{\mu^4 + (a(t) - \tau)^4}, & \text{for } a \geq \tau \\ 0, \text{ for } a < \tau \end{cases}$$

Supplementary Material 2

With the elements described in the previous section we will formulate a model of BMP4 and Vg1 dynamics in chicken embryo cells.

Biological assumptions of the model of BMP4 and Vg1 dynamics

Our model of BMP4 and Vg1 dynamics is based on the following biological assumptions:

A1. Cells in the marginal zone are assumed identical, i.e., they show no differences regarding the molecular mechanisms responsible for the dynamics of BMP4 and Vg1. This assumption implies that molecular interactions shown in the previous diagram take place in every cell of the marginal zone of the embryo. We assume the following spatial arrangement of cells in the marginal zone:

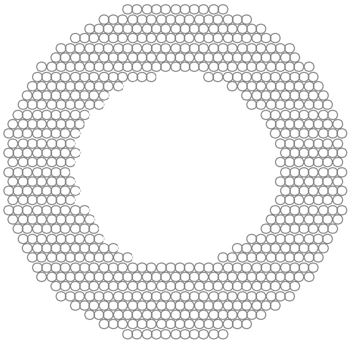

A2. BMP4 and Vg1 have a paracrine mode of action, i.e., they interact with specific receptors located in the membrane of cells.

A3. Vg1 inhibits BMP4 expression.

A4. Experimental results summarized in Figure 1, show that ectopic BMP4 can induce or inhibit *Vg1* in different regions of the embryo. Given that all cells are assumed to be identical (biological assumption A1), such differences must be an emergent property of the embryo as a whole. A simple assumption compatible with these results is that BMP4 induces and activates *Vg1* expression in a dose-dependent manner in every cell of the embryo, a mode of action that has been described for BMP4 in other biological systems [6–8].

Assumptions **A2** and **A3** can be represented by means of the following diagram:

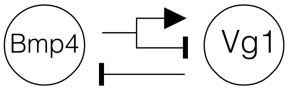

A5. Interactions between BMP4 and Vg1 are mediated by transcription factors. For the sake of simplicity, we will consider only two such factors, labelled as F_B and F_V. We will further assume that mutual effects of BMP4 and Vg result from interactions involving transcription factors F_B and F_V, as summarized in the following figure:

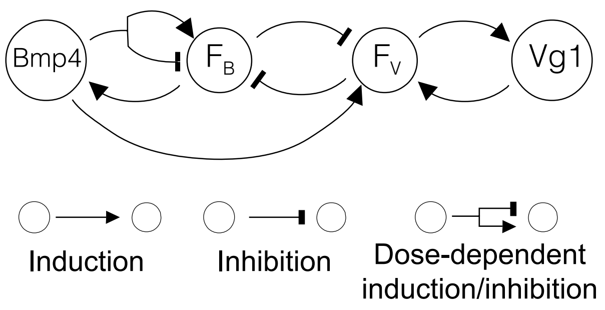

A6. Interactions between BMP4, Vg1, F_B and F_V will be modeled by means of Hill-type function H^+, H^- and $H^\pm$ as described in section SM1.

A7. There is no global processing of information in the embryo. In particular, long-range diffusion of signals has negligible effects. Accordingly, embryo cells have only access to local information in the form of amounts of BMP4 and Vg1 released by each cell and by its neighbors.

A8. BMP4, Vg1, F_B and F_V decay at a certain constant rate.

A9. To determine initial conditions variables B_i, V_i and F_{Vi} are set to zero for every cell i of the embryo. As for transcription factor F_B, we set$F_B = 0$ for half of the cells (chosen at random), and $F_B = 1$ for the rest of the cells.

Scheme of the mathematical model

Biological assumptions enumerated in the previous section are represented in the following figure:

We recall that the dynamics of Bmp4, Vg1, F_B and F_V are identical in all cells of the embryo (assumption **A6**). From this scheme the dynamics of these proteins in each individual cell i can be written as follows:

$$\begin{aligned}
&\text{Intracellular space:}\\
F'_{Bi} &= \text{production controlled by } B_i \text{ and } F_{Vi} - \text{decay}\\
F'_{Vi} &= \text{production controlled by } B_i,\ V_i \text{ and } F_{Bi} - \text{decay}\\
&\text{Extracellular space:}\\
B'_i &= \text{production controlled by } F_{Bi} \pm \text{diffusion} - \text{decay}\\
V'_i &= \text{production controlled by } F_{Vi} \pm \text{diffusion} - \text{decay}
\end{aligned}$$

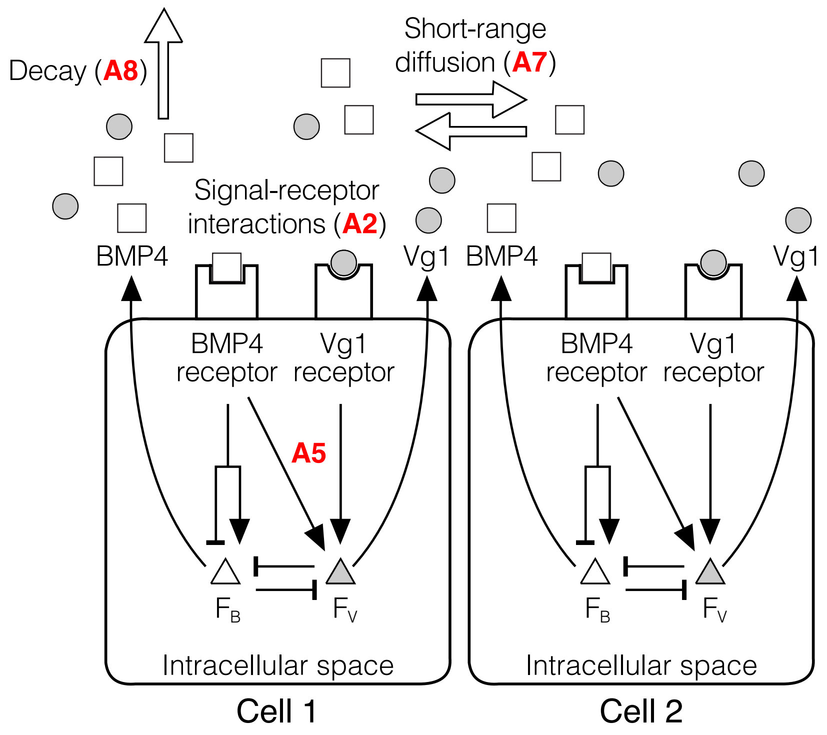

Variables B_i and V_i represent the concentrations of Bmp4 and Vg1 in the extracellular space of the cell. These concentrations are determined by the production of Bmp4 and Vg1 by cell i, the short-range diffusion between neighbor cells and the decay of the signals, assumed to follow a standard first-order kinetics. The interactions of Bmp4 and Vg1 with membrane receptors are assumed proportional to their concentrations, and result in the inhibition or induction of intracellular factors F_B and F_V according to Hill-type functions described above. These factors, in turn, interact with each other to determine the production and secretion of Bmp4 and Vg1 to the extracellular space. Production of BMP4 and Vg1 are assumed to be linear functions of F_B and F_V respectively.

Equations of the model

With the elements introduced in the previous section we are in disposition to write the equations of the model for each cell i:

$$\begin{cases} F'_{Bi} = H^{\pm}(B_i, F_{Bi}) + \dfrac{\sigma_{F_V F_B}\mu^4_{F_V F_B}}{\mu^4_{F_V F_B} + F^4_{Vi}} - \delta_{F_B} F_{Bi} \\ F'_{Vi} = \dfrac{\sigma_{BF_V} B_i^4}{\lambda^4_{BF_V} + B_i^4} + \dfrac{\sigma_{VF_V} V_i^4}{\lambda^4_{VF_V} + V_i^4} + \dfrac{\sigma_{F_B F_V}\mu^4_{F_B F_V}}{\mu^4_{F_B F_V} + F^4_{Bi}} - \delta_{F_V} F_{Vi} \\ B'_i = \alpha_B F_{Bi} + D_B \sum_{j\in N_i}(B_j - B_i) - \delta_B B_i \\ V'_i = \alpha_V F_{Vi} + D_V \sum_{j\in N_i}(V_j - V_i) - \delta_V V_i. \end{cases}$$

where

$$H^{\pm}(B_i, F_{Bi}) = \begin{cases} \dfrac{\sigma_{BF_B}(B_i - \tau_{BF_B})^4}{\lambda^4_{BF_B} + (B_i - \tau_{BF_B})^4} \dfrac{\mu^4_{BF_B}}{\mu^4_{BF_B} + (B_i - \tau_{BF_B})^4} & \text{if } B_i \geq \tau \\ 0 \quad \text{otherwise} \end{cases}$$

Variables B_k and V_k represent the concentrations of Bmp4 and Vg1 in the vicinity of cell k respectively, F_{Bk} and F_{Vk} are the concentrations of transcription factors in cell k, and N_i is the set of neighbor cells of cell i. Parameters $\lambda_{P_1P_2}$ (respectively $\mu_{P_1P_2}$) are the coefficients of Hill functions that describe the induction (respectively the inhibition) of protein P_2 by protein P_1 (see SM1). Parameters $\sigma_{P_1P_2}$ represent the maximum effect (inducer or inhibitor) of protein P_1 on the expression of protein P_2. Parameter δ_P is the decay rate of protein P (for $P = \{B, V, F_B, F_V\}$). Parameters α_B and α_V are the rates of BMP4 and Vg1 production per unit of transcription factors F_B and F_V respectively. Finally, parameters D_B and D_V represent the diffusion coefficients of BMP4 and Vg1 respectively. The values of the parameters used in simulations displayed in figures 2 and 3 are (in suitable units): $\sigma_{BF_B} = 1$, $\tau_{BF_B} = 10$, $\lambda_{BF_B} = 4$, $\mu_{BF_B} = 15$, $\sigma_{F_V F_B} = 1$, $\mu_{F_V F_B} = 2$, $\delta_{F_B} = 0.17$, $\sigma_{BF_V} = 0.1$, $\lambda_{BF_V} = 16.6$, $\sigma_{VF_V} = 1.1$, $\lambda_{VF_V} = 6.6$, $\mu_{F_B F_V} = 2$, $\delta_{F_V} = 0.15$, $\alpha_B = 2.8$, $D_B = 1.95$, $\delta_B = 1.75$, $\alpha_V = 1.5$, $D_V = 1.8$, and $\delta_V = 1.75$.

In each biological setting, the choice of appropriate units and parameter values depends on the availability of significant information about the relevant time and space scales involved. For instance, our simulations reveal that a pattern is formed after 400-500 time units. If one assumes, say that actual patterning unfolds in about three or four hours and that the dominant time scale is that of protein secretion after gene activation, that would yield an estimate for the inverse of that characteristic time (parameters α_B and α_V) between 2 and 3.

We conclude by observing that, in spite of its simplicity, the mathematical model proposed allows to numerically explore the relevance of the biological assumptions made. For instance, changing the value of parameter α_V (see SM2) leads to a different pattern of expression of Vg1 at stage X. In contrast with the normal situation, in which Vg1 is expressed in the posterior region of the embryo, for larger values of α_V, Vg1 becomes uniformly expressed across the marginal zone. This might result in the appearance of multiple axes at later stages of development, and hence to the formation of twins.

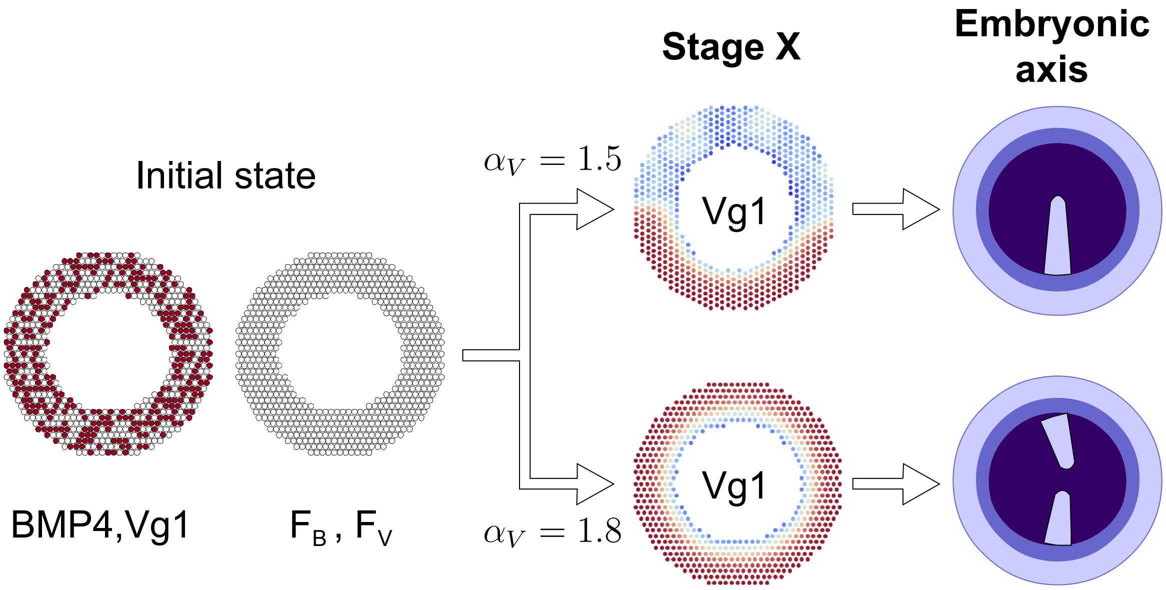

References

1. H. Eyal-Giladi, S. Kochav, From cleavage to primitive streak formation: a complementary normal table and a new look at the first stages of the development of the chick. I. General morphology. *Developmental biology* 49, 321-337 (1976)

2. F. Bertocchini, C. D. Stern, The hypoblast of the chick embryo positions the primitive streak by antagonizing nodal signaling. *Developmental cell* 3, 735-744 (2002)

3. H. Lutz, Sur la production exprimentale de la polyembryonie et de la monstruosit double chez les oiseaux. *Arch. Anat. Microsc. Morphol. Exp* 38, 79-144 (1949).

4. N. T. Spratt, H. Haas, Integrative mechanisms in development of the early chick blastoderm. I. Regulative potentiality of separated part. *J. Exp. Zool.* 145, 97- 137 (1960).

5. F. Bertocchini, C. D. Stern, Gata2 provides an early anterior bias and uncovers a global positioning system for polarity in the amniote embryo. *Development* 139, 4232-4238 (2012); published online EpubNov (10.1242/dev.081901).

6. Suzuki, Y., Yandell, M. D., Roy, P. J., Krishna, S., Savage-Dunn, C., Ross, R. M., ... and Wood, W. B. (1999). A BMP homolog acts as a dose-dependent regulator of body size and male tail patterning in *Caenorhabditis elegans*. *Development*, 126(2), 241-250.

7. James, R. G., and Schultheiss, T. M. (2005). Bmp signaling promotes intermediate mesoderm gene expression in a dose-dependent, cell-autonomous and translation-dependent manner. *Developmental biology*, 288(1), 113-125.

8. Bonilla-Claudio, M., Wang, J., Bai, Y., Klysik, E., Selever, J., and Martin, J. F. (2012). Bmp signaling regulates a dose-dependent transcriptional program to control facial skeletal development. *Development*, 139(4), 709-719.
